# Characterisation of ASD traits among a cohort of children with isolated fetal ventriculomegaly

**DOI:** 10.1038/s41467-023-37242-0

**Published:** 2023-03-21

**Authors:** Vanessa Kyriakopoulou, Alice Davidson, Andrew Chew, Nidhi Gupta, Tomoki Arichi, Chiara Nosarti, Mary A. Rutherford

**Affiliations:** 1 Centre for the Developing Brain, School of Biomedical Engineering and Imaging Sciences, King’s College London, London, UK; 2 Department of Paediatric Neurosciences, Evelina London Children’s Hospital, Guy’s and St Thomas’ NHS Foundation Trust, London, UK; 3 MRC Centre for Neurodevelopmental Disorders, King’s College London, London, UK; 4 Department of Bioengineering, Imperial College London, London, UK; 5 Department of Child and Adolescent Psychiatry, Institute of Psychiatry, Psychology and Neuroscience, King’s College London, London, UK

**Keywords:** Paediatric research, Outcomes research

## Abstract

Fetal ventriculomegaly is the most common antenatally-diagnosed brain abnormality. Imaging studies in antenatal isolated ventriculomegaly demonstrate enlarged ventricles and cortical overgrowth which are also present in children with autism-spectrum disorder/condition (ASD). We investigate the presence of ASD traits in a cohort of children (*n* = 24 [20 males/4 females]) with isolated fetal ventriculomegaly, compared with 10 controls (*n* = 10 [6 males/4 females]). Neurodevelopmental outcome at school age included IQ, ASD traits (ADOS-2), sustained attention, neurological functioning, behaviour, executive function, sensory processing, co-ordination, and adaptive behaviours. Pre-school language development was assessed at 2 years. 37.5% of children, all male, in the ventriculomegaly cohort scored above threshold for autism/ASD classification. Pre-school language delay predicted an ADOS-2 autism/ASD classification with 73.3% specificity/66.7% sensitivity. Greater pre-school language delay was associated with more ASD symptoms. In this study, the neurodevelopment of children with isolated fetal ventriculomegaly, associated with altered cortical development, includes ASD traits, difficulties in sustained attention, working memory and sensation-seeking behaviours.

## Abstract (web summary)

Isolated fetal ventriculomegaly is the most common antenatally-diagnosed brain abnormality. Here, the authors show that isolated fetal ventriculomegaly is associated with autism spectrum disorder traits.

## Introduction

Fetal ventriculomegaly is the most common brain abnormality detected on antenatal ultrasound affecting 1% of fetuses^1^. It is diagnosed when the atrial diameter of either lateral ventricle exceeds 10 mm at any gestation^2^. Approximately 50% of ventriculomegaly cases are associated with additional brain abnormalities (non-isolated ventriculomegaly), the presence of a congenital infection or genetic abnormalities^3^. In the remaining cases, the etiology of the apparently isolated dilatation remains unknown^4–6^. In truly isolated ventriculomegaly (with a negative congenital infection screen and normal genetics) prognosis is currently based on the size of the dilatation, as defined by the atrial diameter measurement on ultrasound or MRI. In these cases, latest studies suggest that outcome is relatively positive, with a > 90% likelihood of normal neurodevelopment in ventriculomegaly of 10 to 12 mm, 75–85% with measurements of 13 to 15 mm and 37% in >15 mm^7–11^. While the atrial diameter only partially predicts outcome, it also does not provide information on the severity or the nature of difficulties in childhood. Therefore, counselling is challenging for clinicians and stressful for parents. Taking into account the high prevalence of the condition, better predicting the severity and the nature of the deficits associated with isolated ventriculomegaly may have important implications in long-term support for the families. Previous studies have focused on dichotomising neurodevelopmental outcomes as favourable or unfavourable, despite a spectrum of outcomes and great variation between individuals. Moving beyond this is crucial, as accurate information regarding the nature of difficulties is essential for counselling parents.

Previous imaging studies suggest that the enlarged ventricles are a marker of altered cortical development with implications for later cognitive and behavioural outcomes^12–15^. We have previously shown that isolated fetal ventriculomegaly is associated with cortical overgrowth during fetal and neonatal life, aberrant development of the white matter tracts and an increased risk of language delay at 2 years of age^13,14^. Of particular relevance, ventriculomegaly and cortical overgrowth have also been widely described in children with autism spectrum disorder/condition (ASD) while language development deficits are a known early sign of ASD^16–22^. However, there are currently no published studies systemically assessing ASD traits in a cohort of children with antenatally-diagnosed isolated ventriculomegaly. Some previous studies have reported incidental cases of ASD although these were not systematically assessed within their study protocols^10,23–26^.

The aim of this study was to investigate the long-term outcome and presence of ASD traits in a cohort of children with isolated fetal ventriculomegaly and the relationship between in-utero brain imaging metrics and long-term neurodevelopment.

In this work, we show that isolated fetal ventriculomegaly is associated with ASD traits, difficulties in sustained attention and working memory and sensation-seeking behaviours.

## Results

### Cohorts

Demographic information for both cohorts are summarised in Tables 1 and 2. Participation rates for the pre-school and primary school age assessments and reasons for non-participation for both cohorts are presented in the Supplementary information (Supplementary Fig.1 and Supplementary Fig.2). In the control cohort, 33 children with a normal fetal brain MR assessment were invited to take part in the study developmental follow-up, 10 children (30%) were assessed at a mean age 5.4 ± 0.8 years. In the ventriculomegaly cohort, 60 children with an antenatal diagnosis of isolated ventriculomegaly were invited to take part in the study developmental follow-up at 2 years and then at school age. 24 children (40%) were assessed at a mean age 5.8 ± 1.2 years. Four children (16%) already had a clinical diagnosis of ASD prior to attending the assessment. One child received a clinical ASD diagnosis following participation in the study. We did not collect information about any diagnosis received after participation in the research study. 23 out of 24 children were attending mainstream school and one child was enrolled in an ASD-specialist school.

**Table 1:** Child’s clinical characteristics

Child Demographics	Control	Ventriculomegaly
Total n	10	24
Sex (m/f)	6 / 4	20 / 4
Atrial diameter	6.8 mm (4–8.7)	12.1 mm (10.1–15.6)
Severity of ventriculomegaly	-	12 mild/ 10 moderate / 2 severe
Laterality of ventriculomegaly	-	19 unilateral / 5 bilateral
GA at scan	29.8 weeks (22.1–37)	29.1 weeks (21.7–37)
GA at birth	40.4 weeks (38.3–42.4)	39.5 weeks (32.6–42.1)
Age at assessment	5.4 years (4.3–6.6)	5.8 years (3.9–8.2)
Age at early language assessment	23.8 months (12–35) (*n* = 9)	23.9 months (12-28) (*n* = 23)
Prior clinical diagnosis of ASD	0	4 (16%)

Mean and ranges are presented for GA at scan, GA at birth and age at assessments. *GA* gestational age.

**Table 2:** Parental socio-demographic and economic characteristics

Parental Demographics	Control	Ventriculomegaly
Maternal education attainment		
GCSE	0%	17%
A-Levels	0%	4%
College	10%	17%
Higher Education	90%	62%
Household income		
<£20,000	10%	29%
£20,000 - £29,999	10%	4%
£30,000 - £39,999	0%	0%
£40,000 - £59,999	20%	4%
£60,000 - £79,999	0%	21%
£80,000 - £99,999	20%	25%
£100,000 - £149,999	20%	13%
> £149,999	20%	4%

### Neuropsychological assessment

#### ADOS-2

**Fig. 1:**
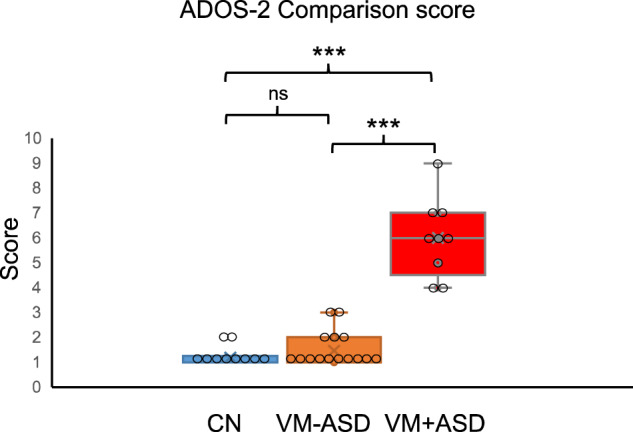
ADOS-2 Comparison scores of study sub-cohorts. The ADOS-2 Comparison score was significantly higher in the VM + ASD sub-cohort compared to the CN cohort (*p* < 0.0001) and compared to the VM-ASD sub-cohort (*p* < 0.0001). There was no significant difference in the ADOS-2 Comparison score between the VM-ASD and control cohort (*p* = 0.482). ns, *p* = 0.482, ***p < 0.001. Box plots indicate median (middle line), mean (x), 25th, 75th percentile (box) and 5th and 95th percentile (whiskers) as well as outliers (single points). The individual data points have been overlaid on the graph. Statistical analysis: Kruskal–Wallis, two-sided, Bonferroni correction applied. CN *n* = 10, VM-ASD *n* = 15, VM + ASD *n* = 9. CN Control, VM + ASD Ventriculomegaly and ADOS-2 autism/ASD classification, VM-ASD Ventriculomegaly and ADOS-2 non-spectrum classification. Source data are provided as a Source Data file.

The ADOS-2 was completed by all attending children in the control and ventriculomegaly cohorts. All children in the control cohort received scores below the threshold for autism/ASD. In the ventriculomegaly cohort, 9 out of 24 children (37.5%) scored above the threshold to meet the criteria for autism/ASD. The severity range spanned low to high (low = 2, moderate = 6, high = 1). There was a significant relationship between a diagnosis of ventriculomegaly and a classification of ADOS-2 autism/ASD (*p* = 0.024, chi square = 5.1, df = 1). The ADOS-2 Comparison score was significantly higher in the ventriculomegaly cohort (*p* = 0.04) compared to the control cohort. Furthermore, the ADOS-2 Comparison score was significantly higher in the VM + ASD sub-cohort (*p* < 0.0001) but not in the VM-ASD when compared to the control cohort (*p* = 0.482) (Fig. 1). All assessment results are summarized in Table 3 and the assessment scores of study sub-cohorts are shown in Supplementary Information (Supplementary Fig.3 and Supplementary Fig.4).

**Table 3:** Assessment results

Assessments	CN			VM			CN vs VM	VM -ASD			VM + ASD			CN vs VM-ASD	CN vs VM + ASD
		*n* = 10			*n* = 24				*n* = 15			*n* = 9			
	(mean ± SD)			(mean ± SD)				(mean ± SD)			(mean ± SD)				
ADOS-2 Comparison score	1.2	±	0.4	2.9	±	2.1	***p*** = **0.04**	1.7	±	0.7	5.6	±	1.1	NS	***p*** < **0.0001**
Preschool language delay n (%)	0	(0%)		9	(39%)			3	(20%)		6	(66.6%)			
Full Scale IQ (WPPSI-IV)	114	±	13	107	±	20	NS	114	±	19	93	±	14	NS	NS
Tracking accuracy (Track-it) (% successful trials)	89%			81%			***p*** = **0.04**	78%			87%			NS	NS
Working memory (Track-it) (%successful trials)	97%			88%			***p*** = **0.02**	88%			88%			NS	***p*** = **0.009**
Adaptive Skills (Vineland-II)															
Adaptive Behaviour	108	±	9	104	±	15	NS	107	±	11	99	±	19	NS	NS
Daily Living Skills	104	±	8	103	±	14	NS	105	±	10	99	±	18	NS	NS
Behaviour (CBCL)															
Total behaviour problems	46.4	±	8.4	44.3	±	12.0	NS	41.3	±	14.6	51.0	±	9.0	NS	NS
Internalising	46.5	±	9.0	46.7	±	11.0	NS	42.6	±	8.4	55.4	±	10.9	NS	NS
Externalising	47.1	±	9.5	43.9	±	11.4	NS	41.7	±	9.3	48.4	±	13.9	NS	NS
Sensory processing (SSP-2)															
Tactile Sensitivity	32.8	±	1.3	32.0	±	3.8	NS	32.7	±	2.4	30.4	±	5.4	NS	NS
Taste/Smell Sensitivity	16.7	±	3.9	17.7	±	3.6	NS	17.9	±	3.3	17.4	±	4.1	NS	NS
Movement sensitivity	14.0	±	1.1	14.5	±	1.1	NS	14.7	±	0.7	14.0	±	1.6	NS	NS
Under-responsive/Seeks Sensation	29.2	±	2.7	28.1	±	5.3	NS	28.9	±	3.6	26.6	±	7.6	NS	NS
Auditory filtering	25.4	±	3.2	24.6	±	5.0	NS	26.1	±	3.1	21.7	±	6.6	NS	NS
Low Energy/weak	29.6	±	0.9	28.2	±	3.0	NS	28.0	±	3.3	28.7	±	2.4	NS	NS
Visual/Auditory Sensitivity	21.5	±	3.1	22.3	±	3.0	NS	22.7	±	1.9	21.4	±	4.5	NS	NS
Executive function (BRIEF-P)															
Global executive composite	49.9	±	6.3	51.2	±	14.4	NS	46.0	±	8.4	61.6	±	17.9	NS	NS
Inhibit	50.5	±	4.5	49.9	±	12.2	NS	45.7	±	8.3	58.1	±	14.4	NS	NS
Shift	46.3	±	8.7	49.4	±	10.2	NS	45.6	±	6.5	57.0	±	11.9	NS	NS
Emotion Control	49.0	±	9.1	54.5	±	14.5	NS	50.4	±	10.7	62.7	±	17.3	NS	NS
Working memory	50.5	±	6.7	51.6	±	15.1	NS	47.5	±	9.0	59.7	±	20.6	NS	NS
Plan/Organise	52.5	±	11.2	50.5	±	13.2	NS	46.9	±	9.4	57.9	±	16.4	NS	NS
Movement/Co-ordination (Little DCDQ)															
Total score	71.3	±	2.7	70.4	±	6.8	NS	71.5	±	4.5	68.0	±	9.7	NS	NS

*CN* control cohort, *VM* Ventriculomegaly cohort, *VM-ASD* Ventriculomegaly and ADOS-2 non-spectrum classification, *VM* *+* *ASD* Ventriculomegaly and ADOS-2 autism/ASD classification, *NS* no significance, *p* > 0.05. Statistical analysis for ADOS-2 Comparison score, BRIEF-P, CBCL, Little DCDQ and SSP-2: Kruskal–Wallis, two-sided, Bonferroni correction applied. Statistical analysis for Full Scale IQ, Track-it and Vineland-III: ANOVA, two-sided, Bonferroni correction applied. *P* values <0.05 are highlighted in bold.

#### Pre-school language development

**Fig. 2:**
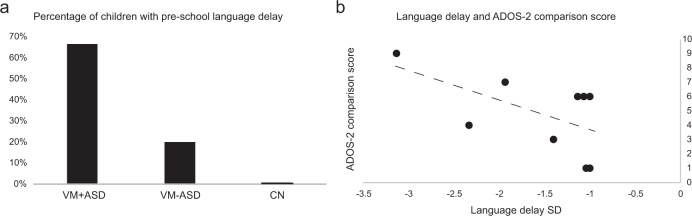
Pre-school language delay and ADOS-2 classification and comparison score. **a** Percentage of children with pre-school language delay in the study sub-cohorts.CN Control, VM + ASD Ventriculomegaly and ADOS-2 autism/ASD classification, VM-ASD Ventriculomegaly and ADOS-2 non-spectrum classification. **b** there was a significant correlation between the degree of pre-school language delay and the ADOS-2 Comparison score (Spearman’s −0.658, *p* = 0.027, two-sided). SD standard deviation. Source data are provided as a Source Data file.

In the control cohort, 9 out of 10 children had a formal assessment of their language development at pre-school age. None of the 9 children had language delay. In the ventriculomegaly cohort, 23 out of 24 children had a formal assessment of their language development at pre-school age. 9 of the 23 children displayed language delay. Six out of those 9 children subsequently scored within the autism/ASD classification at the ADOS-2 assessment. Within the ventriculomegaly cohort, there was a significant relationship between the presence of language delay and the classification of ADOS-2 autism/ASD (*p* = 0.022, chi square = 5.2, df = 1). The percentage of children with language delay for each sub-cohort is presented in Fig. 2a.

The presence of language delay at pre-school age predicted an ADOS-2 autism/ASD classification with a specificity of 73.3% and a sensitivity of 66.7%. Additionally, in the children with language delay, there was a significant correlation between the degree of pre-school language delay and the ADOS-2 Comparison score (Spearman’s −0.658, *p* = 0.027) (Fig. 2b).

#### WPPSI-IV

All children in the control cohort had Full scale IQ scores within 1 SD of the mean. In the ventriculomegaly cohort, one child was unable to complete the WPPSI-IV because they were not verbal, 19 children had IQ scores in the normal range (above 1 SD) while 4 children had IQ scores within the 1 SD to 2 SD range. There was no significant difference in Full scale IQ scores between cohorts (*p* = 0.336).

#### Track-it

Children in the ventriculomegaly cohort had significantly lower scores in Tracking Accuracy (F = 4.35, *p* = 0.04) and in Memory Accuracy (F = 5.61, *p* = 0.02), when correcting for the age at assessment.

#### Parental questionnaires

The T-scores from the parental questionnaires in probing their child’s adaptive skills, behaviour, sensory processing, executive function and movement/co-ordination, are presented below. There was no significant difference in the T-scores for the above domains between cohorts. A higher percentage of children in the ventriculomegaly cohort had scores reaching the clinical cut-off in sensory processing compared to controls (Table 4). There was a significant association between sensation-seeking behaviours and the presence of ventriculomegaly (*p* = 0.04, chi-square = 4.02, df = 1).

**Table 4:** The percentage of children with scores reaching the standardised clinical cut-off

Developmental domain	CN	VM	VM-ASD	VM + ASD
Adaptive Skills (Vineland-II)				
Communication	0%	0%	0%	0%
Daily Living Skills	0%	8%	7%	11%
Socialisation	0%	8%	7%	22%
Motor skills	10%	17%	13%	22%
Adaptive Behaviour	0%	4%	0%	11%
Sensory processing (SSP-2)				
Tactile Sensitivity	0%	21%	20%	22%
Taste/Smell Sensitivity	30%	17%	13%	22%
Movement sensitivity	10%	8%	0%	22%
Under-responsive/Seeks Sensation*	10%	42%	40%	22%
Auditory filtering	10%	25%	20%	33%
Low Energy/weak	0%	21%	27%	11%
Visual/Auditory Sensitivity	0%	8%	0%	22%
Executive function (BRIEF-P)				
Inhibit	0%	13%	0%	33%
Shift	0%	8%	0%	22%
Emotion Control	10%	21%	13%	33%
Working memory	0%	13%	7%	22%
Plan/Organise	10%	13%	7%	22%
Global Executive Composite	0%	13%	0%	33%
Behaviour (CBCL)				
Emotionally Reactive	10%	8%	0%	22%
Anxious/Depressed	10%	8%	0%	22%
Somatic Complaints	10%	4%	0%	11%
Withdrawn	0%	8%	0%	22%
Sleep Problems	10%	4%	0%	11%
Attention Problems	0%	0%	0%	0%
Aggressive Behaviour	10%	8%	0%	22%
Internalising	10%	8%	0%	22%
Externalising	0%	4%	0%	11%
Movement/co-ordination (Little DCDQ)				
Total score	10%	8%	0%	22%

The * indicates a significant association with ventriculomegaly.

#### Neurological examination

All children in the control cohort had a normal neurological examination. In the ventriculomegaly cohort, one child had a benign hand tremor and one child showed coordination and balance abilities within the lower range for his age. The rest of the children performed within the normal range.

#### Demographics

Within the control cohort, there were no cases of maternal depression or family history of ASD. One child in the control cohort had a half-sibling with an ASD diagnosis. There was no reported family history of ASD in the children in the ventriculomegaly cohort. There were three cases with a history of maternal depression (VM + ASD *n* = 1, VM-ASD *n* = 2), although it is unknown as to whether these were prenatal diagnoses. All inter-cohort and correlation analysis were repeated after removing these 3 cases; there was no significant change in the results. In the ventriculomegaly cohort, 8 parents expressed concerns about their child’s development, in 5 out of 8 their child also scored above the ADOS-2 ASD threshold. In the control cohort, one parent expressed concerns about their child’s development. There was no significant difference in maternal education attainment (*p* = 0.118, chi-square = 2.44, df = 1) or household income (*p* = 0.151, chi-square = 5.29, df = 3) between cohorts. All inter-cohort and correlation analyses were repeated covarying for maternal education attainment and household income.

### Imaging

#### Atrial diameter

The atrial diameter of the control and ventriculomegaly cohorts were 6.8 ± 1.4 mm (4–8.7 mm) and 12.1 ± 1.6 mm (10.1–15.6 mm) respectively (see below). Demographics about the ventriculomegaly sub-cohorts are presented in Table 5. While the proportion of moderate and bilateral ventriculomegaly cases was skewed towards the VM + ASD cohort, there was no significant difference in the severity of ventriculomegaly (*p* = 0.16, chi-square = 1.97, df = 1) or laterality (*p* = 0.496, chi-square = 0.46, df = 1) between the VM-ASD and VM + ASD sub-cohorts.

**Table 5:** Demographic and clinical characteristics of the ventriculomegaly sub-cohorts

Demographics	VM -ASD	VM + ASD
Total n	**15**	**9**
Sex (m/f)	11 / 4	9 / 0
Atrial diameter	12 mm (10.3–15.6)	12.3 mm (10.1–15.3)
Severity of ventriculomegaly	9 mild / 5 moderate / 1 severe	3 mild / 5 moderate / 1 severe
Laterality of ventriculomegaly	13 unilateral / 2 bilateral	6 unilateral / 3 bilateral
GA at birth	39.6 weeks (36.4–41.9)	39.3 weeks (32.6–42.1)
Birthweight centile	48th (8th-90th)	60th (15th-93rd)

Mean and ranges are presented for the atrial diameter and GA at birth. The median and range of centiles are presented for the birthweight centiles. *GA* gestational age.

#### Brain volumetric analysis

Cortical volumes corrected for gestational age and head circumference were significantly larger in the ventriculomegaly cohort compared to controls, with a mean difference of 10.6% (F = 10.9, *p* = 0.002). There was no significant difference in total ventricular (F = 0.11, *p* = 0.737) or cortical volume (F = 0.28, *p* = 0.281) between the two ventriculomegaly sub-cohorts (VM + ASD and VM-ASD), when corrected for gestational age at scan and head circumference.

#### Relationship between brain measurements and assessment metrics

There was no correlation between atrial diameter, total ventricular or cortical volume and the ADOS-2 metrics in the control or ventriculomegaly cohorts. In the ventriculomegaly cohort, there was a correlation between total ventricular volume (corrected for GA and fetal head circumference) and the Block Design score (Spearman’s −0.499, *p* = 0.005), Picture Memory (Spearman’s −0.403, *p* = 0.03), Full Scale IQ (Spearman’s −0.396, *p* = 0.03), sustained attention (Spearman’s −0.486, *p* = 0.006), movement sensory sensitivity (Spearman’s −0.491, *p* = 0.006) and opposition defiant disorder scores (Spearman’s −0.374, *p* = 0.04). Higher ventricular volumes were associated with a worse performance in the above domains. However, these correlations did not withstand the Bonferroni correction for multiple comparisons. After correcting for maternal education attainment and household income, there was a significant correlation between total ventricular volume and the block design score (Spearman’s −0.478, *p* = 0.024) and aggressive behaviour (Spearman’s 0.451, *p* = 0.035). These correlations did not withstand the Bonferroni correction for multiple comparisons.

## Discussion

In this study we have shown that 37.5% of children with isolated fetal ventriculomegaly had ADOS-2 scores that met the threshold for an ASD diagnosis. Our findings expand on those of previous neurodevelopmental outcome studies which have reported incidental cases of ASD, although this has never been systematically studied^10,23–26^. Moreover, language delay is a common early precursor of ASD and has been frequently reported in children with isolated ventriculomegaly^11,14,24,27,28^. In this study, pre-school language delay predicted a score within the ADOS-2 autism/ASD classification with a specificity of 73.3% and a sensitivity of 66.7%. The severity of language delay correlated with the level of ASD symptoms. This may provide an opportunity for early identification and targeted support for the most at-risk children.

Children with isolated ventriculomegaly received significantly lower scores in the sustained attention Track-it task, both in Tracking accuracy and Working Memory. However, it was not possible to determine whether this was due to inattention or working memory underperformance (or both) as these executive function skills are closely intertwined. When comparing the performance on the WPPSI-IV working memory task, there were no significant inter-group differences, with only 2 children in the ventriculomegaly cohort having scores below 1 SD from the mean. This apparent inconsistency may be explained by the design of the two working memory tasks. The Track-it working memory task has a higher cognitive load because of the greater time elapsing between stimulus presentation and recall, as well as the presence of distractors, compared to the WPPSI-IV working memory task, where the recall phase occurs immediately after the presentation phase, with no distractors. In the ventriculomegaly cohort, the total ventricular volume correlated negatively with the WPPSI-IV working memory task scores, with children with larger ventricles performing worse. This may indicate that there is no single sustained attention/working memory deficit, but a more complex interplay of concurrently developing underlying processes of executive function. Atad-Rapoport et al.^29^ assessed children at 9–11 years of age and showed that children with unilateral ventriculomegaly had significantly lower scores in sustained attention and parental-reported executive function abilities but not in working memory abilities. Leitner et al.^30^ reported that 21% of children with isolated ventriculomegaly had developmental difficulties and lower achievement scores. These were attributed to deficits in attention and working memory. Future studies utilizing eye-tracking could provide more information on the individual executive function components and how these relationships are altered in the presence of ventriculomegaly.

Children with ventriculomegaly were also more likely to have sensation-seeking behaviours. More specifically, there was a significant association between sensory seeking behaviours and the presence of ventriculomegaly, with almost half of the children in the ventriculomegaly cohort scoring within the clinical range. While this may be expected in children with ASD, these associations were detected in both sub-cohorts, with and without an ADOS-2 ASD classification. Sensory seeking severity also correlated with ventricular size.

Children with sensory seeking behaviours actively create opportunities to increase sensory input during tasks and appear active, excitable, and continuously engaged in their environments. Children with larger ventricles also performed worse in the Block Design task, which involves spatial visualisation abilities and visual-motor dexterity, and the Matrix Reasoning task assessing visual information processing and abstract reasoning. Interestingly, in neonates with antenatally-diagnosed ventriculomegaly, we have previously identified white matter microstructure alterations in the posterior thalamic radiation, which contains the optic radiations^14^.

We found no difference in Full Scale IQ between the cohorts. Our study is in agreement with previous reports that have found no difference in general intelligence test performance between children with isolated ventriculomegaly and controls^24,25,29,30^. Impairments in adaptive behaviours and intellectual abilities are common in children with ASD^31,32^. The majority of children in the VM + ASD sub-cohort, had mean IQ scores and reported adaptive behaviours that fell within the normal range. This suggests they may represent an etiological subtype of ASD; from a clinical perspective, children with ventriculomegaly may have a favourable outcome within the ASD spectrum. A detailed neurological examination was performed on every participant and in agreement to previous studies there were no motor impairments in children with ventriculomegaly^14,24,25,29^. There are some reports of delayed motor development in a small proportion of children although it is unclear whether this is a persistent clinical abnormality or a delay in achieving developmental milestones^33,34^. The presence of additional CNS and non-CNS abnormalities, use of isolated screening tools and no physical neurological examination, may further obscure these results.

The lack of a significant predictive relationship between ventricular or cortical size and long-term development may indicate that the outcome of children with ventriculomegaly is not governed by the size of the lateral ventricles or cortex in a mono-dimensional way, rather these deviations represent the tip of the iceberg. The neural connections formed during fetal and neonatal life are further established during the first postnatal years and refined by environmental input to form the basis for the emergence of higher-order social, language and cognitive networks and behaviour^35^. There was a higher proportion of children with antenatally-diagnosed moderate (≥12 mm) and bilateral ventriculomegaly in the VM + ASD sub-cohort. However, this did not reach statistical significance. Previous studies indicate that laterality (bilateral) and ventricular size (>12 mm) are factors associated with a worse neurodevelopmental outcome^9–11,30,36^. Our cohort size is small and interpretations should be made with caution. Further confirmation by larger studies is warranted.

The cohort of children assessed are part of a larger study and the results of detailed fetal and neonatal brain imaging have been previously published^13,14^. The brain phenotype of fetal ventriculomegaly includes ventricular and cortical enlargement and aberrant development of the white matter tracts. Similarly, in this smaller but representative cohort we have shown a significant cortical enlargement in the fetal scans of the children enrolled in the follow-up part of the study. We hypothesise that cortical overgrowth seen in fetal ventriculomegaly, may occur secondary to disruption in the regulation of cell proliferation and apoptosis and subsequent altered brain connectivity, which may explain the high risk of ASD in these children. Ventriculomegaly, cortical overgrowth and disordered white matter microstructure have also been described in young children with ASD^16–20,37^. A comprehensive meta-analysis by Sacco et al^38^. of 8310 participants, spanning from toddlers to adults, showed brain and head circumference overgrowth in ASD compared to controls. This was most prominent in early childhood. A retrospective ultrasound study by Bonnet-Brilhault et al.^39^, showed that head overgrowth was present in the second and third trimesters of pregnancy in fetuses who were diagnosed with ASD later in childhood. Our study provides additional in-vivo evidence that brain and head overgrowth associated with isolated ventriculomegaly begins prenatally in a subtype of ASD. Assessing ventricular size is part of the standard sonographic fetal examination internationally and may therefore provide a categorical, radiologically identifiable, and clinically detectable prenatal risk factor of ASD.

Fetal ventriculomegaly affects 1% of fetuses and 50% of those are isolated cases. Based on our study results, VM + ASD might occur in 0.2% of all fetuses. The prevalence of ASD in children in England is 1.76%^40^. Therefore, the estimated contribution of VM + ASD in the ASD population could reach 12%.

Evidence from postmortem, genome, animal models, molecular and cellular studies indicate altered development in ASD, with prenatal origins^35^. An excess of prefrontal cortex neurons (67% increase) has been found at autopsy in children with autism (*n* = 7) compared to controls (*n* = 6) which could underlie the volume increases in cerebral gray matter reported by Courchesne et al.^41^ The authors suggested that such an increase in neuronal numbers can only have occurred during fetal life as there is no other time during brain development during which such a large excess of cortical neurons is generated. iPS (induced pluripotent stem cells) derived neural progenitor cells from toddlers with ASD and brain overgrowth, display excess proliferation which correlates with the degree of brain overgrowth quantified on MRI^42^. Decreased elimination of neural processes, including apoptosis, axonal pruning, and dendritic degeneration, as well as increased neurogenesis have also been suggested to occur in autism^43^. Developmental apoptosis occurring during the fetal period is a determining factor in the number of persistent subplate neurons seen in the adult brain^44,45^. An increase in the number of these surviving descendants of the fetal subplate has been shown in postmortem tissue of children and adults with ASD, indicating an abnormal fate in this population^46^. Apoptosis is an integral part of cortical development, initially related to morphogenesis, proliferation and regulation of progenitor size and during the late fetal and postnatal period to synaptogenesis. This process by which exuberant neuronal branches and connections are removed is essential for establishing the functional organisation of circuitry. Atypical patterns of connectivity may be associated with neurodevelopmental disorders. The cause and underlying mechanism of isolated ventriculomegaly are unknown, thus there are no animal models engineered specifically for the condition. In addition, there is no availability of post-mortem samples to investigate the underlying molecular and cellular pathology.

There are some limitations to be noted. The control cohort is relatively small (*n* = 10) and has a lower ratio of boys/girls compared to the ventriculomegaly cohort. Furthermore, all children with ventriculomegaly and an ASD classification were boys. Therefore, future studies with larger number of participants and sex-matched datasets are needed to corroborate these findings. Another possible limitation is that only a sub-cohort of children invited to participate took part in the study follow-up. It could be argued that participation was driven by parental concerns about their child’s development, thus inflating the results. Concerns regarding their child’s development were expressed by parents in both cohorts. Concerns may also have been generated following the results of the early-age assessment, with parents of children with language delay more likely to attend the school age assessments. However, the attendance rates for the school age assessment of children with and without early language delay were similar (60% vs 50% respectively) (Supplementary Fig.1 and Supplementary Fig.2). Lastly, while there was no difference in maternal education attainment levels between cohorts, the percentage of participants with a higher education qualification (controls 90%, ventriculomegaly 63%) was higher than the percentage observed in the general population in England (41%)^47^.

The major strength of the study lies in the combined assessment of language development and subsequent administration of the ADOS-2, which is considered the gold standard for the assessment of ASD symptoms. While the ADOS-2 is the gold-standard to accurately access and diagnose ASD, an isolated administration is not sufficient for a diagnosis. For this reason, we differentiate between clinically diagnosed ASD and ADOS-2 classified ASD. A clinical diagnosis of ASD should be made based on a comprehensive assessment of developmental history, cognitive and communicative functioning, and observation of ASD symptoms using multiple diagnostic and cognitive instruments. It would therefore be inappropriate for us to make any assumptions. Nevertheless, the prevalence of clinically diagnosed ASD in our study cohort is 16% (Results/Cohort) which is considerably higher than the prevalence of ASD in children in England; 1.76% with a male to female ratio of 3:1^40^. A proportion of the children in the ventriculomegaly cohort scored above clinical cut-off scores in the ADOS-2 but did not have a clinical diagnosis of ASD prior to the assessment. While the age of assessment in this study is similar to the age of ASD diagnosis in the general population (5 years), there are factors that may have influenced the age of clinical diagnosis^48,49^. There is a lack of standardised clinical guidelines in the UK for recommended follow-up following a diagnosis of ventriculomegaly. In the absence of any parental or teacher concerns, a child would be unlikely to have received a formal assessment outside of this research study. Additionally, the majority of children in the VM + ASD sub-cohort, had mean IQ scores and reported adaptive behaviours that fell within the normal range and were therefore less likely to have significant difficulties in their daily lives; thus their parents’ may have been less likely to request additional support or an assessment. It is possible that children may have received an ASD diagnosis after the study assessment, however we are unable to take this information into account as there were no scheduled communications with the families as part of the study protocol.

The selection of a homogenous ventriculomegaly cohort, in the absence of additional brain abnormalities, infection or genetic disorders has been crucial in interpreting outcome results. A normal karyotype and a lack of clinical features excluded a genetic diagnosis in our study. However, with advances in genetic testing, further investigations such as microarray and genome sequencing may provide additional information about this cohort and their susceptibility in developing ASD. A large number of ASD risk genes are involved in neurogenesis, proliferation and early synaptogenesis, show a peak expression during the prenatal period and in brain regions that are altered in ASD (BrainSpan RNA-Seq dataset, http://www.brainspan.org/)^35^.

There has been a clear need for data describing the average outcome of children antenatally-diagnosed isolated ventriculomegaly^50,51^. We have shown that the long-term neurodevelopment includes ASD traits, difficulties in sustained attention, working memory and sensation-seeking behaviours. General intelligence test performance and adaptive skills were largely unaffected while there was a lack of motor impairments. 23 out of 24 children were attending mainstream school and one child was enrolled in an ASD-specialist school. Our study has demonstrated an association between the most common developmental fetal brain anomaly, isolated ventriculomegaly, diagnosable by antenatal US or MR imaging, and ASD traits. The results from this small study may improve counselling for families and aid early identification, support, and intervention. Further research is warranted to confirm our findings in a larger population.

## Methods

Ethical approval was granted by the NHS Research Ethics Committee (ethics No. 07/H0707/105) and informed consent obtained from all participants at each visit. The research study was conducted in accordance to the approved study protocol and complied with all relevant ethical regulations. The study included children that had undergone a fetal brain MRI at the Robert Steiner MRI Unit at the Hammersmith Hospital, London between November 2009 and August 2013. Study visits took place at the Hammersmith Hospital and Guy’s and St Thomas’ Hospital in London. Participants were reimbursed for their travel expenses only.

### Cohorts

The ventriculomegaly cohort presented in this study, is part of a larger cohort^13^ and includes fetuses with a diagnosis of isolated ventriculomegaly on MRI during November 2009 – August 2013. Clinical referrals for the assessment of the fetal brain using MRI were received following a diagnosis of ventriculomegaly on the routine clinical ultrasound performed at around 20 weeks gestation. Fetuses with additional CNS and non-CNS abnormalities, positive infection screening and chromosomal abnormalities, fetal growth restriction (FGR), twin pregnancies, maternal drug use or poor image quality were excluded from this study. Fetuses with brain MR appearances consistent with atrophy of the surrounding tissue or with increased intraventricular pressure were also excluded following clinical reporting.

The control cohort presented in this study is also part of a larger cohort^13,52^. For the purposes of this study, the above larger control dataset was reviewed, and control fetuses were selected and invited to participate in the follow-up study. The control cohort matched the ventriculomegaly cohort in the following variables:

- Geographical location: all children were recruited from Greater London and surrounding areas.
- MRI findings: The MRI control cohort is comprised of children with normal sized ventricles.
- Socio-economic status, which included maternal education attainment and household income.
- Overlap in time period: Children in the MRI control cohort were assessed during the same time period (December 2015–November 2018), thus minimising potential historical data bias.

The control cohort included healthy pregnant volunteers with a normal fetal brain MRI assessment. Participants were excluded from the control cohort if there were abnormal findings on fetal MRI, inadequate MR image quality, delivery complications, congenital malformations or infection, chromosomal abnormality, twin pregnancy, preterm delivery (≤36 weeks gestational age (GA)), abnormal clinical neonatal examination, abnormal findings on neonatal MR examination, or abnormal neurodevelopmental examination at either one or two years of age. Fetal GA was estimated from a first trimester dating ultrasound scan.

### Assessments

The battery of assessments at school age included the Autism Diagnostic Observation Schedule-2 (ADOS-2), the Wechsler Preschool and Primary Scales of Intelligence IV (WPPSI-IV), a neurological examination and the Track-it computer task. These were administered on the same day. Parental questionnaires were completed prior to the child’s assessment.

### Autism spectrum symptoms

The ADOS-2 was used for the observational assessment of ASD^53^. As described in the ADOS-2 assessment manual, the ADOS-2 Overall Total score is Module specific and was converted to the ADOS-2 Classification (autism, autism spectrum, non-spectrum) and the ADOS-2 Comparison Score. The ADOS-2 Comparison Score represents the level of autism spectrum-related symptoms (1–2 = minimal-to-no evidence, 3–4 = low, 5–7 = moderate, 8–10 = high). The ADOS-2 Classification and Comparison scores are comparable between individuals and across Modules and were used for all statistical analysis in this study.

The ADOS-2 was performed and scored by a highly trained assessor. Scoring was performed during the session and subsequently from a video recording, and also by a second independent assessor during the assessment and from the video recording. Any discrepancies (i.e. lack of an exact agreement between the two assessors in ADOS-2 algorithm scores) were discussed with a certified ADOS-2 trainer and a final score was agreed amongst the two assessors and the trainer. The second assessor and third assessors (ADOS-2 trainer) were blinded to the MRI results. Both ADOS-2 assessors were trained and certified research reliable in all modules (Pearson Clinical Assessment). Research reliability was maintained throughout the duration of the study by attending bi-monthly research reliability maintenance training at the Institute of Psychiatry, Psychology and Neuroscience, King’s College London.

### Cognitive development

The WPPSI-IV was used to assess cognitive development^54^. IQ was evaluated from the child’s performance on tasks assessing verbal comprehension, visual spatial, fluid reasoning, working memory and processing speed. The Full scale IQ score has a mean of 100 and a standard deviation of 15. Subtest scaled scores have a mean of 10 and a standard deviation of 3. Full scale IQ and subtest scaled scores were age adjusted.

### Sustained attention

The Track-It computer task was used to assess sustained attention^55^. The task was performed at a viewing distance of 50 cm and was controlled by the assessor. All children were asked to visually follow a single target object moving among 2 different distractor objects. Target and distractor objects were randomly selected from a pool of unique objects (e.g. yellow star, purple diamond). The objects moved randomly on a 3 × 3 grid. At the end of each trial, all the objects disappeared, and the child was asked to point to the grid the target was in when it disappeared. The task consisted of 1 practice and 10 test trials. At the beginning of each trial, the child viewed the objects in a static image with the target object marked by a red circle. The child was asked to confirm the target object they would be visually following. At the end of each trial, all 9 objects were presented, and the child was asked to identify the target they were following. The trial length was 10.9 s, and the object speed was 500 pixels per second and at 30 frames per second. The responses to the 10 test trials for the final position of the target object were averaged to produce a Tracking Accuracy score for each child while the responses to the memory check questions were averaged to produce a Memory Accuracy score.

### Neurological examination

The neurological clinical status of each child was assessed by an experienced pediatrician (AC, NG, TA). This included a detailed assessment of the upper and lower limb muscle tone, power, reflexes and sensation; cerebellar function; gait; and cranial nerve function.

### Parental questionnaires

The battery of standardised questionnaires completed by the parents included the Behaviour Rating Inventory of Executive Function Preschool version (BRIEF-P) (executive function metrics: Global executive function composite, inhibit, shift, emotion control, working memory, plan/organise)^56^, Short Sensory Profile-2 (SSP-2) (sensory processing metrics: tactile sensitivity, movement sensitivity, under-responsive/seeks sensation, auditory filtering, low energy/weak, visual/auditory sensitivity)^57^, Child Behaviour Checklist (CBCL) (behaviour metrics: total behaviour problems, internalising, externalising)^58^, Little Developmental Coordination Disorder Questionnaire – Canadian (Little DCDQ-CA) (movement and co-ordination metric: total motor)^59^ and the Vineland-II (adaptive behaviour metrics: adaptive behaviour, daily living skills)^60^. The T-scores from each questionnaire were used for inter-cohort comparisons. Use of reference normative data from each questionnaire allowed for the adjustment of age at assessment and sex for each participant. A score reaching the pre-defined clinical cut-offs, indicated a delay or difficulties in the specified developmental area. Maternal education attainment (level of highest qualification) and household income data were recorded.

### Pre-school language development

Pre-school assessments (2 years of age) were conducted using the Griffiths Mental Development Scales or the Bayley-III Scales of Infant and Toddler Development^61,62^. These include a language assessment component. Language delay was defined by a language composite score below 1 SD or an expressive language scaled score below 1SD. Due to the different assessment tools used, the scores between the assessment modalities are not comparable, we therefore present the proportion of children that displayed language delay and also the standard deviation from the mean.

### Fetal MRI acquisition and image post-processing

All children had a fetal brain MRI scan either following maternal recruitment as a healthy volunteer (control cohort) or following a clinical referral for ventriculomegaly on antenatal ultrasound (ventriculomegaly cohort). Details of image acquisition and processing have been previously described^13,52^. Briefly, fetal scans were performed on a 1.5Tesla MRI System. The atrial diameter, head circumference and volumetric measurements of the cortex and lateral ventricles were performed on motion corrected slice-to-volume reconstructed super-resolution images as previously described^52,63,64^.

### Statistics

Statistical analysis was performed using SPSS (version 26, SPSS IBM). Analysis of variance (ANOVA) was used for continuous variables that were normally distributed and Kruskal–Wallis for non-parametric comparisons. Bonferroni correction was used to account for multiple comparisons. Spearman’s correlation coefficient was used to investigate correlations between brain volumetric measures and outcome measures. The Pearson’s Chi-square test was used to assess relationships between nominal variables. Assessment metrics were corrected for age at assessment and sex. Ventricular and cortical volumes were corrected for gestational age at scan and head circumference. Head circumference was selected to correct for head size differences between participants and sexes^52^. To distinguish between behaviours associated with the presence of ASD, the ventriculomegaly cohort was further sub-divided according to their ADOS-2 classification (VM-ASD, VM + ASD).

### Reporting summary

Further information on research design is available in the Nature Portfolio Reporting Summary linked to this article.

## Supplementary information

Supplementary Information

Reporting Summary

## Data Availability

Source data for Figs. 1 and 2 are provided in the Source Data file. The imaging data, individual developmental assessment scores and source data for Tables 1, 2, 3 and 4 are not openly available as we do not have participant permission to share this data. Source data are provided with this paper.

## Source data

Source Data

## Code availability

No new algorithms were generated as part of this study. Previously published software were used to generate the analyses as detailed in the Methods section and are freely and publicly available:

1. Track-it! computer task for assessing sustained attention in children. This is a free and publicly available software. https://github.com/JohnDickerson/TrackIt.

2. Fetal MR image motion correction and reconstruction were performed using the SVR methodology. This is a free and publicly available code https://github.com/SVRTK/SVRTK.

3. Fetal MR volumetric quantification was performed using DrawEM v.1.1. This is a free and publicly available code. https://github.com/MIRTK/DrawEM/tree/fetal.
