# Supplementary material for: Characterisation of ASD traits among a cohort of children with isolated fetal ventriculomegaly
Source: Nat Commun. 2023 Mar 21;14:1550. doi: 10.1038/s41467-023-37242-0 (PMC10027681; doi:10.1038/s41467-023-37242-0)
Supplement: Supplementary file 1 — Supplementary Information [file 41467_2023_37242_MOESM1_ESM.pdf]

Supplementary information for Characterisation of ASD traits among a cohort of children with isolated fetal ventriculomegaly

Contents

Figure S1. Participation rates in the ventriculomegaly cohort during the study

Figure S2. Participation rates in the control cohort during the study

Figure S3. Assessment scores of study sub-cohorts

Figure S4. Distribution of assessment scores of study sub-cohorts

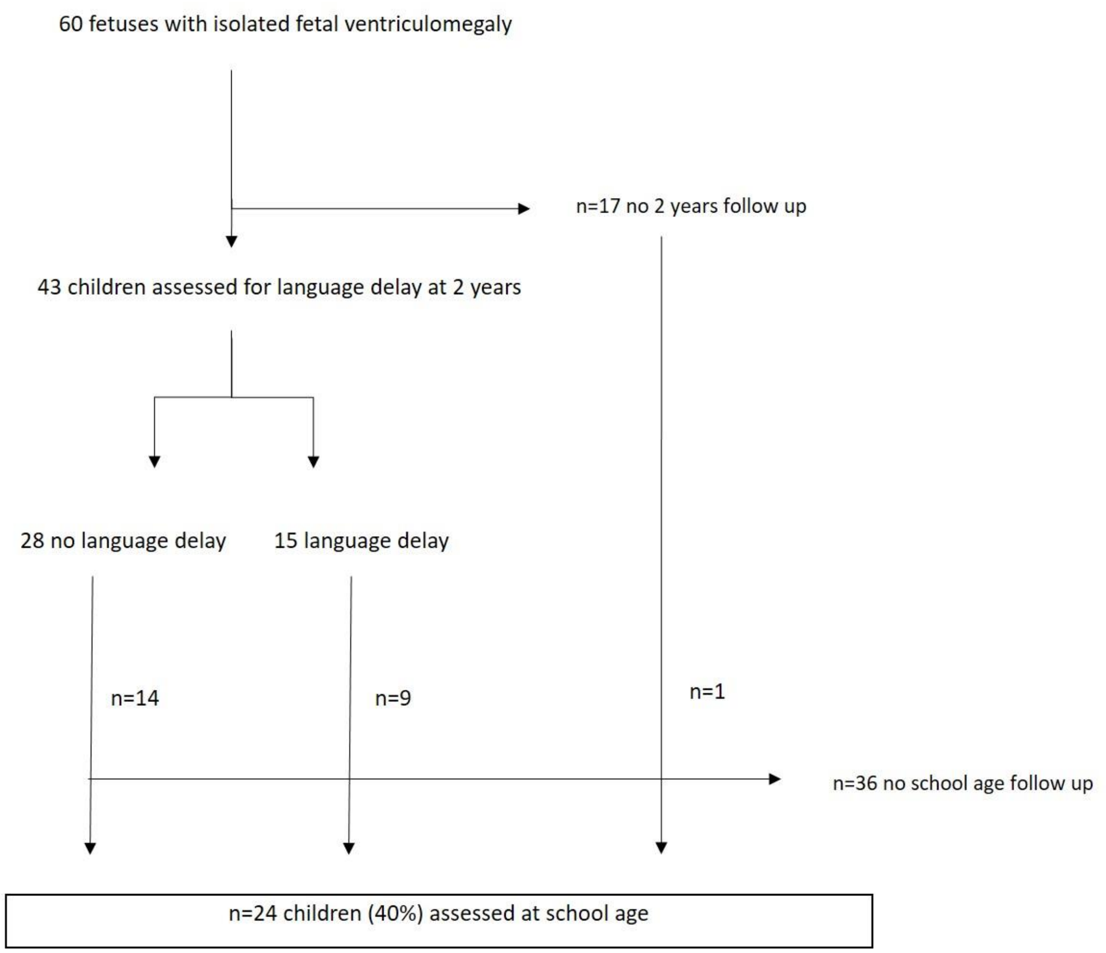

Figure S1. Participation rates in the ventriculomegaly cohort during the study

The reasons for non-participation at primary school age in the ventriculomegaly cohort were as following:

24 No response

2 Declined ("too far to travel")

3 Declined ("no longer interested")

3 Declined ("lack of time due to busy schedule")

4 Accepted but subsequently cancelled the appointment

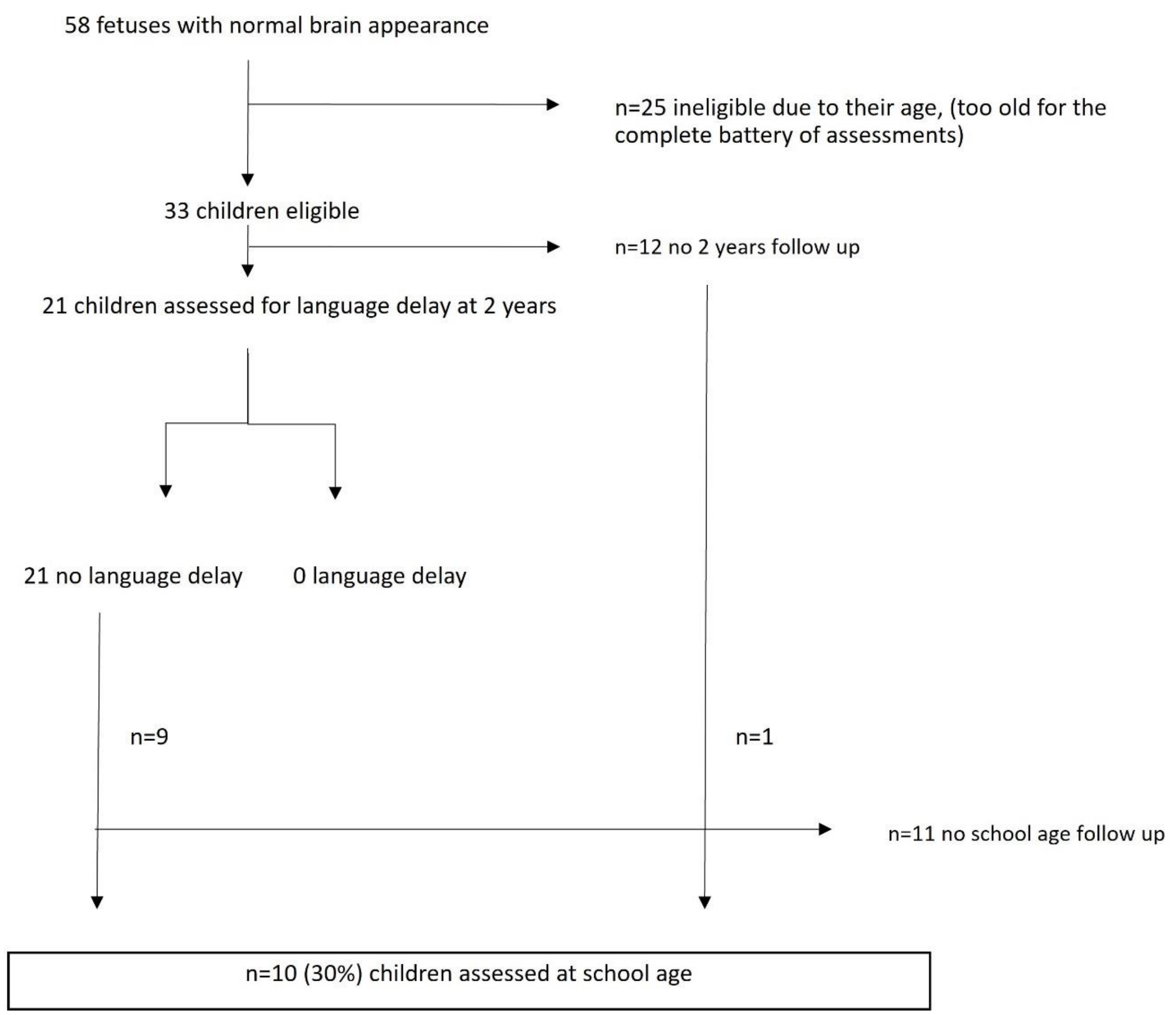

Figure S2. Participation rates in the control cohort during the study

The reasons for non-participation at primary school age in the control cohort were as following:

17 No response

2 Declined ("no longer interested")

3 Declined ("lack of time due to busy schedule")

1 Accepted but subsequently cancelled the appointment

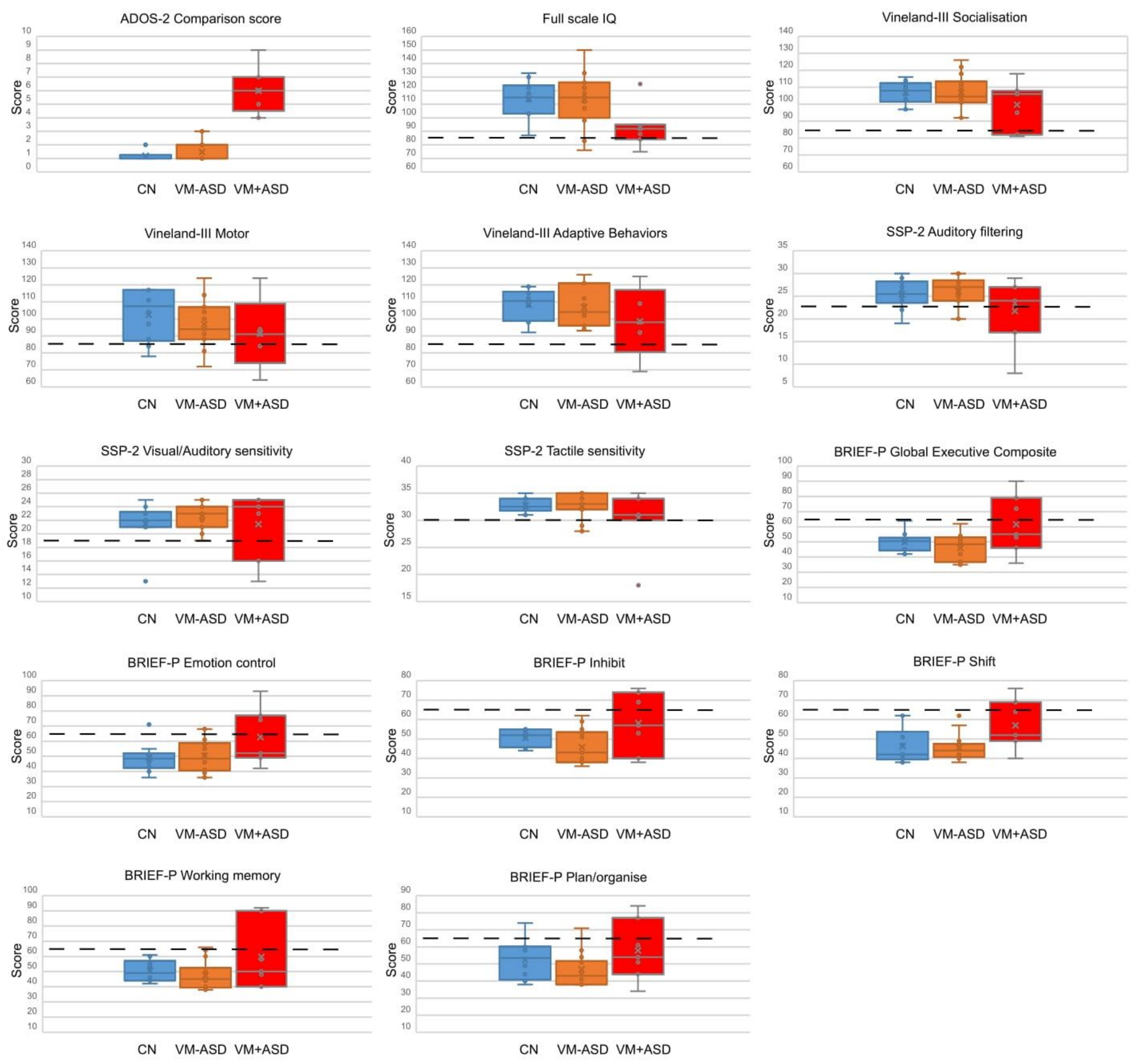

Figure S3. Assessment scores of study sub-cohorts

Assessment scores for IQ, ADOS-2, Vineland-III, SSP-2 and BRIEF-P.

The dotted line represents the point of -1SD (Full Scale IQ, Vineland-III) or clinical cut-off (SSP-2, BRIEF-P). Box plots indicate median (middle line), mean (x), 25th, 75th percentile (box) and 5th and 95th percentile (whiskers) as well as outliers (single points). For ADOS-2 Comparison Score, Vineland-III Socialisation, Vineland-III Motor, Vineland-III Adaptive behaviours, SSP-2 Auditory filtering, SSP-2 Visual/Auditory sensitivity, SSP-2 Tactile sensitivity, BRIEF-P Global Executive Composite, BRIEF-P Emotion control, BRIEF-P Inhibit, BRIEF-P Shift, BRIEF-P Working memory and BRIEF-P Plan/Organise: CN n=10, VM-ASD n=15, VM+ASD n=9. For Full Scale IQ: CN n=10, VM-ASD n=15, VM+ASD n=8.

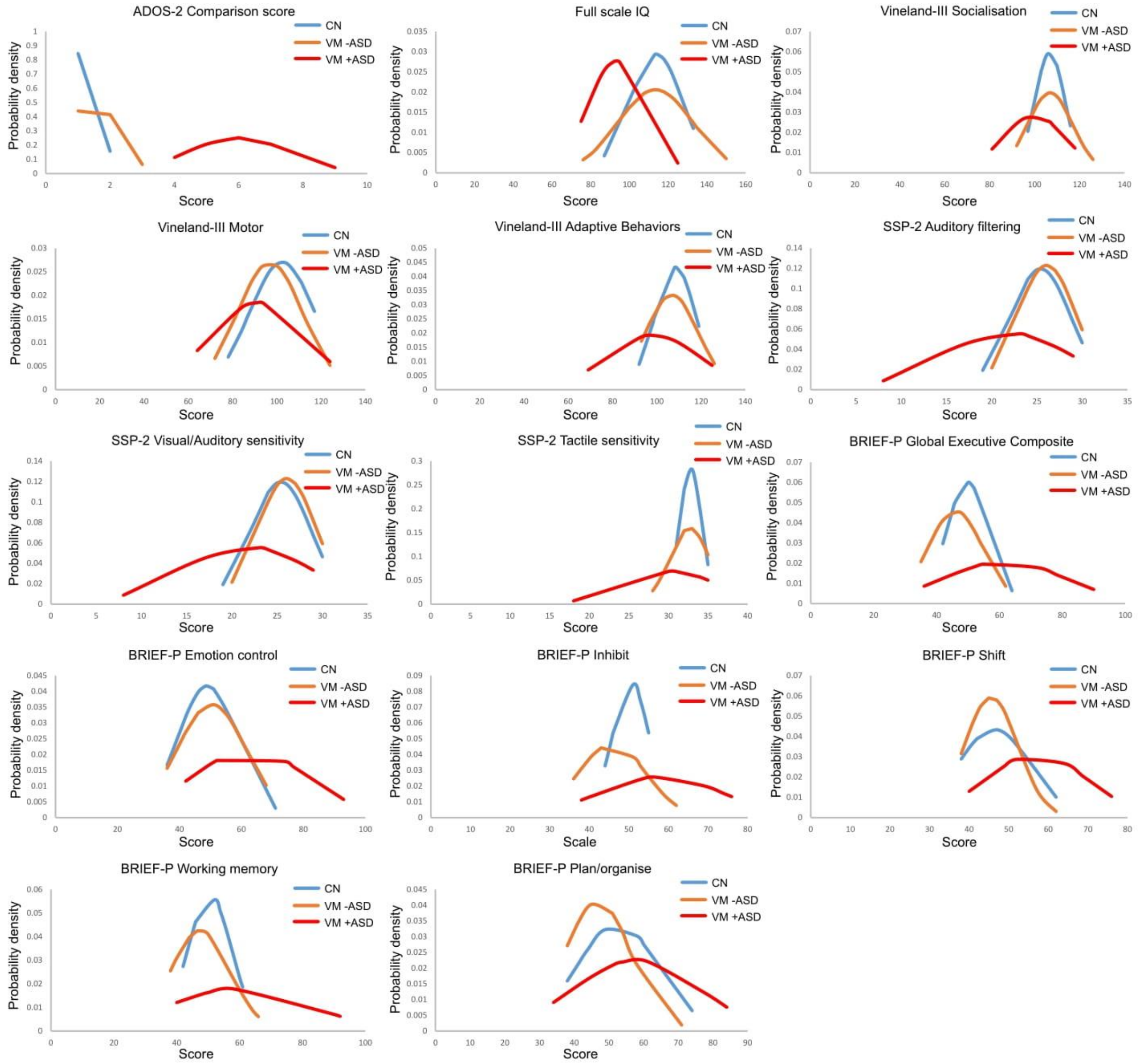

Figure S4. Distribution of assessment scores of study sub-cohorts

Distribution of assessment scores for IQ, ADOS-2, Vineland-III, SSP-2 and BRIEF-P.
