# Supplementary material for: Characterisation of ASD traits among a cohort of children with isolated fetal ventriculomegaly
Source: Nat Commun. 2023 Mar 21;14:1550. doi: 10.1038/s41467-023-37242-0 (PMC10027681; doi:10.1038/s41467-023-37242-0)
Supplement: Supplementary file 2 — Reporting Summary [file 41467_2023_37242_MOESM2_ESM.pdf]

nature portfolio

Corresponding author(s): Dr Vanessa Kyriakopoulou

Last updated by author(s): Jan 3, 2023

Reporting Summary

Nature Portfolio wishes to improve the reproducibility of the work that we publish. This form provides structure for consistency and transparency in reporting. For further information on Nature Portfolio policies, see our Editorial Policies and the Editorial Policy Checklist.

Statistics

For all statistical analyses, confirm that the following items are present in the figure legend, table legend, main text, or Methods section.

n/a	Confirmed	
☐	☒	The exact sample size (*n*) for each experimental group/condition, given as a discrete number and unit of measurement
☒	☐	A statement on whether measurements were taken from distinct samples or whether the same sample was measured repeatedly
☐	☒	The statistical test(s) used AND whether they are one- or two-sided<br>*Only common tests should be described solely by name; describe more complex techniques in the Methods section.*
☐	☒	A description of all covariates tested
☐	☒	A description of any assumptions or corrections, such as tests of normality and adjustment for multiple comparisons
☐	☒	A full description of the statistical parameters including central tendency (e.g. means) or other basic estimates (e.g. regression coefficient) AND variation (e.g. standard deviation) or associated estimates of uncertainty (e.g. confidence intervals)
☐	☒	For null hypothesis testing, the test statistic (e.g. F, t, r) with confidence intervals, effect sizes, degrees of freedom and P value noted<br>*Give P values as exact values whenever suitable.*
☒	☐	For Bayesian analysis, information on the choice of priors and Markov chain Monte Carlo settings
☒	☐	For hierarchical and complex designs, identification of the appropriate level for tests and full reporting of outcomes
☐	☒	Estimates of effect sizes (e.g. Cohen's *d*, Pearson's *r*), indicating how they were calculated

Our web collection on statistics for biologists contains articles on many of the points above.

Software and code

Policy information about availability of computer code

Data collection	Track-it! computer task for assessing sustained attention in children. This is a free and publicly available software. https://github.com/JohnDickerson/TrackIt Publication: FISHER, A., THIESSEN, E., GODWIN, K., KLOOS, H. & DICKERSON, J. 2013. Assessing selective sustained attention in 3- to 5-year-old children: evidence from a new paradigm. J Exp Child Psychol, 114, 275-94.
Data analysis	1. Fetal MR image motion correction and reconstruction were performed using SVR according to: JIANG, S., XUE, H., GLOVER, A., RUTHERFORD, M., RUECKERT, D. & HAJNAL, J. V. 2007. MRI of moving subjects using multislice snapshot images with volume reconstruction (SVR): application to fetal, neonatal, and adult brain studies. IEEE Trans Med Imaging, 26, 967-80. Latest version available https://github.com/SVRTK/SVRTK<br><br>2. Fetal MR volumetric quantification was performed using DrawEM v.1.1. Publication: MAKROPOULOS, A., GOUSIAS, I. S., LEDIG, C., ALJABAR, P., SERAG, A., HAJNAL, J. V., EDWARDS, A. D., COUNSELL, S. J. & RUECKERT, D. 2014. Automatic whole brain MRI segmentation of the developing neonatal brain. IEEE Trans Med Imaging, 33, 1818-31. This is a free and publicly available code. https://github.com/MIRTK/DrawEM/tree/fetal<br><br>3. IBM SPSS Statistic (version 26, SPSS IBM). https://www.ibm.com/uk-en/products/spss-statistics

For manuscripts utilizing custom algorithms or software that are central to the research but not yet described in published literature, software must be made available to editors and reviewers. We strongly encourage code deposition in a community repository (e.g. GitHub). See the Nature Portfolio guidelines for submitting code & software for further information.

Data

Policy information about availability of data

All manuscripts must include a data availability statement. This statement should provide the following information, where applicable:

- Accession codes, unique identifiers, or web links for publicly available datasets
- A description of any restrictions on data availability
- For clinical datasets or third party data, please ensure that the statement adheres to our policy

The data that support the findings of this study are not openly available as they contain information that could compromise research participant privacy.

Human research participants

Policy information about studies involving human research participants and Sex and Gender in Research.

Reporting on sex and gender	The sex (biological attribute) of the participants was determined at birth and recorded in the neonatal delivery summary. Gender data were not collected as part of this study. Neurodevelopmental assessment metrics were corrected/adjusted for sex.
Population characteristics	"See above"
Recruitment	Recruitment during the antenatal period:<br><br>Ventriculomegaly cohort: Our department is a tertiary centre and receives clinical referrals for the assessment of the fetal brain using MRI, from hospitals in Greater London and surrounding areas. Clinical referrals were received following a diagnosis of ventriculomegaly on the routine clinical ultrasound performed at around 20 weeks gestation. At this stage, the pregnant women were invited to participate in the research study. The ventriculomegaly cohort presented in this study, is part of a larger cohort (Kyriakopoulou et al., 2014) and includes fetuses with a diagnosis of isolated ventriculomegaly on MRI during November 2009 - August 2013. Fetuses with additional CNS and non-CNS abnormalities, positive infection screening and chromosomal abnormalities, fetal growth restriction (FGR), twin pregnancy maternal drug use or poor image quality were excluded from this study. Fetuses with brain MR appearances consistent with atrophy of the surrounding tissue or with increased intraventricular pressure were also excluded following clinical reporting.<br><br>Control cohort: Participants in the control cohort were also recruited during their routine clinical ultrasound timepoint, performed at around 20 weeks gestation. Pregnant women attending their routine appointment were provided information about the study and invited to participate. The control cohort included healthy pregnant volunteers with a normal fetal brain MRI assessment. Participants were excluded from the control cohort if there were abnormal findings on fetal MRI, inadequate MR image quality, delivery complications, congenital malformations or infection, chromosomal abnormality, twin pregnancy, preterm delivery (≤36 weeks gestational age (GA)), abnormal clinical neonatal examination, abnormal findings on neonatal MR examination, or abnormal neurodevelopmental examination at either one or two years of age.<br><br>Fetal GA was estimated from a first trimester dating ultrasound scan.<br><br>The control cohort presented in this study is also part of a larger cohort (Kyriakopoulou et al., 2017, Kyriakopoulou et al., 2014). For the purposes of this study, the above larger control dataset was reviewed, and control fetuses were selected and invited to participate in the follow-up study. The control cohort matched the ventriculomegaly cohort in the following variables:<br>- Geographical location: all children were recruited from Greater London and surrounding areas.<br>- MRI findings: The MRI control cohort is comprised of children with normal sized ventricles.<br>- Socio-economic status, which included maternal education and household income.<br>- Overlap in time period: Children in the MRI control cohort were assessed during the same time period (December 2015-November 2018), thus minimising potential historical data bias.<br><br>Continued participation in the study follow-up:<br>Only a sub-cohort of children took part in the study follow-up. To evaluate self-selection bias, all parents that attended the school-age assessment were asked if they had any concerns regarding their child's development, prior to receiving the results of the assessment. It could be argued that participation was driven by parental concerns about their child's development, thus inflating the results. In the ventriculomegaly cohort, 30% of parents reported concerns regarding their child's development, in 2/3 of cases their child had scored above the ADOS-2 ASD threshold. One parent in the control cohort expressed concerns about their child's development. Concerns may also have been generated following the results of the early-age assessment, with parents of children with language delay more likely to attend the school age assessments. However, the attendance rates for the school age assessment of children with and without early language delay were similar (60% vs 50% respectively).
Ethics oversight	Ethical approval was granted by the NHS Research Ethics Committee (ethics No. 07/H0707/105).

Note that full information on the approval of the study protocol must also be provided in the manuscript.

Field-specific reporting

Please select the one below that is the best fit for your research. If you are not sure, read the appropriate sections before making your selection.

☐ Life sciences ☒ Behavioural & social sciences ☐ Ecological, evolutionary & environmental sciences

For a reference copy of the document with all sections, see nature.com/documents/nr-reporting-summary-flat.pdf

Behavioural & social sciences study design

All studies must disclose on these points even when the disclosure is negative.

Study description

Case-control quantitative study. Participants were recruited into the study during the antenatal period (fetal brain MRI scan) and their neurodevelopmental outcome was assessed up at 2 years and primary school age timepoints.

Research sample

A representative cohort was recruited during the antenatal period including all clinical referrals received from multiple hospitals across a large urban setting (Greater London) and surrounding areas, during a time period of ~3.5 years and including all severities of ventricular enlargement (mild, moderate, severe).

The cohorts at the school-age assessment timepoint included 24 children with antenatally-diagnosed isolated ventriculomegaly (ventriculomegaly cohort) and 10 children with normal-sized ventricles (control cohort), based on in-utero MRI. The ventriculomegaly cohort was recruited following clinical referrals received from across Greater London and surrounding areas (UK) during November 2009 - August 2013. Participants in the control cohort were recruited from the antenatal clinics during the same time period.

Demographics

Ventriculomegaly cohort
Sex: 20 males / 4 females
Gestational age at scan: 29.1 weeks
Age at early language assessment: 23.9 months
Age at primary school assessment: 5.8 years

Control cohort
Sex: 6 males / 4 females
Gestational age at scan: 29.8 weeks
Age at early language assessment: 23.8 months
Age at primary school assessment: 5.4 years

Sampling strategy

Sampling method: convenience. All patients with a clinical referral and diagnosis of isolated ventriculomegaly, received during November 2009 - August 2013, were invited to participate in the research study. This is a teriary centre receiving clinical referrals from across Greater London and surrounding areas (UK). Participants in the control cohort were recruited from the antenatal clinics during the same time period. Sample sizes were pre-determined from the previous publication characterising the brain phenotype of these cohorts (Kyriakopoulou et al., 2014).

Data collection

The instruments used for data collection were:
1. Fetal brain image acquisition: 1.5T MRI scanner.
2. MR image analysis: computer.
3. Neurodevelopmental assessments: observations and scoring were recorded using pen/paper and video-recording (only for ADOS-2 and neurological examination). Parental questionnaires were scored using pen/paper.
4. Track-it computer game: computer.

Only the participants (child and parent) and the researchers were present during data collection.

The second assessor and third assessors (ADOS-2 trainer) were blinded to the MRI results. All assessors were blinded to any clinical diagnosis prior to the assessment.

Timing

Data collection for the study started in November 2009 and finished in November 2018.

Data exclusions

Pre-established exclusion criteria were applied. These parameters may have an effect on brain development and/or later neurodevelopment.

Ventriculomegaly cohort exclusion criteria:
1. Additional CNS and non-CNS abnormalities
2. Positive infection screening and chromosomal abnormalities
3. Fetal growth restriction (FGR)
4. Twin pregnancy

5. Maternal drug use
6. Poor image quality
7. Brain MR appearances consistent with atrophy of the surrounding tissue
8. Brain MR appearances consistent with increased intraventricular pressure.

Control cohort exclusion criteria:
1. Abnormal findings on fetal MRI
2. Inadequate MR image quality
3. Delivery complications
4. Congenital malformations
5. Positive maternal infection
6. Chromosomal abnormality
7. Twin pregnancy
8. Preterm delivery (<36 weeks gestational age (GA)),
9. Abnormal clinical neonatal examination
10. Abnormal findings on neonatal MR examination, or
11. Abnormal neurodevelopmental examination at either one or two years of age.

Non-participation

Isolated ventriculomegaly cohort:
60 fetuses with isolated ventriculomegaly were recruited
24 children (40%) were assessment at primary school age

The reasons for non-participation at primary school age in the ventriculomegaly cohort were as following:
24 No response
2 Declined ("too far to travel")
3 Declined ("no longer interested")
3 Declined ("lack of time due to busy schedule")
4 Accepted but subsequently cancelled the appointment

Normal control cohort:
33 normal control fetuses were selected for comparison with the above clinical cohort
10 children (30%) were assessment at primary school age

The reasons for non-participation at primary school age in the control cohort were as following:
17 No response
2 Declined ("no longer interested")
3 Declined ("lack of time due to busy schedule")
1 Accepted but subsequently cancelled the appointment

Randomization

Allocation was based on the results of the brain MRI assessment. Participants were recruited into the ventriculomegaly following a diagnosis of isolated ventriculomegaly on MRI. The control cohort included healthy pregnant volunteers with a normal fetal brain MRI assessment.

Reporting for specific materials, systems and methods

We require information from authors about some types of materials, experimental systems and methods used in many studies. Here, indicate whether each material, system or method listed is relevant to your study. If you are not sure if a list item applies to your research, read the appropriate section before selecting a response.

Materials & experimental systems

n/a	Involved in the study	
☒	☐	Antibodies
☒	☐	Eukaryotic cell lines
☒	☐	Palaeontology and archaeology
☒	☐	Animals and other organisms
☐	☒	Clinical data
☒	☐	Dual use research of concern

Methods

n/a	Involved in the study	
☒	☐	ChIP-seq
☒	☐	Flow cytometry
☐	☒	MRI-based neuroimaging

Clinical data

Policy information about clinical studies

All manuscripts should comply with the ICMJE guidelines for publication of clinical research and a completed CONSORT checklist must be included with all submissions.

Clinical trial registration	Ethical approval was granted by the NHS Research Ethics Committee (ethics No. 07/H0707/105). This study does not have an intervention and therefore does not fall under the ICMJE definition of a clinical trial.
Study protocol	Not publicly available.
Data collection	Data collection took place in Hammersmith Hospital (NHS) and St Thomas' Hospital (NHS) during the time period November 2009 - November 2018.
Outcomes	Outcomes were assessed at pre-school (2 years) and at primary school age. (>4 years).<br><br>1. Pre-school assessments (2 years of age) were conducted using the Griffiths Mental Development Scales or the Bayley-III Scales of Infant and Toddler Development. Only language delay was reported.<br>2. The battery of assessments at school age (>4 years) included the Autism Diagnostic Observation Schedule-2 (ADOS-2), the Wechsler Preschool and Primary Scales of Intelligence IV (WPPSI-IV), a neurological examination and the Track-it computer task. The battery of standardised questionnaires completed by the parents included the Behaviour Rating Inventory of Executive Function Preschool version (BRIEF-P) (executive function metrics: Global executive function composite, inhibit, shift, emotion control, working memory, plan/organise), Short Sensory Profile-2 (SSP-2) (sensory processing metrics: tactile sensitivity, movement sensitivity, under-responsive/seeks sensation, auditory filtering, low energy/weak, visual/auditory sensitivity), Child Behaviour Checklist (CBCL) (behaviour metrics: total behaviour problems, internalising, externalising), Little Developmental Coordination Disorder Questionnaire – Canadian (Little DCDQ-CA) (movement and co-ordination metric: total motor) and the Vineland-II (adaptive behaviour metrics: adaptive behaviour, daily living skills).

Magnetic resonance imaging

Experimental design

Design type	Structural MRI
Design specifications	Not task-based.
Behavioral performance measures	Not-task based.

Acquisition

Imaging type(s)	Structural
Field strength	1.5 Tesla
Sequence & imaging parameters	T2-weighted Single-Shot Turbo-Spin Echo (ssTSE) was acquired using the following scanning parameters: time repetition = 15 000 ms, time echo = 160 ms, slice thickness of 2.5 mm, slice overlap of 1.5 mm, flip angle = 90°.
Area of acquisition	Brain

Diffusion MRI ☐ Used ☒ Not used

Preprocessing

Preprocessing software	Brain reconstruction: Multislice Snapshot Images With Volume Reconstruction (SVR)<br>Brain masking: BET implemented in FSL toolbox.<br>Automated segmentation of brain structures: DrawEM v1.1<br>Manual editing/refinement of brain structures: ITK-SNAP v2.2
Normalization	The intensity of the images was automatically normalized to the same range as the part of the DrawEM pipeline. In addition, all images were bias-corrected using the N4 algorithm in ITK (as part of the DrawEM pipeline).
Normalization template	Spatial normalization template for segmentation included manually segmented T2 images and atlases of the neonatal brain as described in MAKROPOULOS, A., GOUSIAS, I. S., LEDIG, C., ALJABAR, P., SERAG, A., HAJNAL, J. V., EDWARDS, A. D., COUNSELL, S. J. & RUECKERT, D. 2014. Automatic whole brain MRI segmentation of the developing neonatal brain. IEEE Trans Med Imaging, 33, 1818-31. https://github.com/MIRTK/DrawEM/tree/fetal
Noise and artifact removal	This is part of the SVR methodology, no additional artifact removal or filtering was applied.
Volume censoring	Each dataset was visually inspected in terms of reconstruction success and image quality by an experienced researcher.

Statistical modeling & inference

Model type and settings	Multivariate analysis. Fixed factors: cohort.
Effect(s) tested	ANOVA was used for inter-cohort comparison of MRI and neurodevelopmental assessment metrics. Kruskal-Wallis was used for non-parametric comparisons.

Specify type of analysis: ☐ Whole brain ☒ ROI-based ☐ Both

Anatomical location(s)	Anatomical location and volumetric quantification were performed using an automated software and followed by manual refinement by a researcher with expertise in fetal neuroanatomy. The total volume of the lateral ventricles was defined as the volume of both left and right lateral ventricles, including the choroid plexus but excluding the third and fourth ventricles, the cavum septum pellucidum (CSP), and vergae. Cortical volume represents the total cerebral cortical gray matte.
Statistic type for inference (See Eklund et al. 2016)	*Specify voxel-wise or cluster-wise and report all relevant parameters for cluster-wise methods.*
Correction	Bonferroni correction for multiple comparisons was applied.

Models & analysis

n/a	Involved in the study
☐	☒ Functional and/or effective connectivity
☒	☐ Graph analysis
☐	☒ Multivariate modeling or predictive analysis

Functional and/or effective connectivity	Spearman’s correlation coefficient was used to investigate correlations between continuous MRI measures and neurodevelopmental outcome measures. The Pearson’s Chi-square test was used to assess relationships between nominal variables.
Multivariate modeling and predictive analysis	Dependent variables: MRI metrics, neurodevelopmental assessment metrics.<br>Covariates: Ventricular and cortical volumes were corrected for gestational age at scan and head circumference. Head circumference was selected to correct for head size differences between participants and sexes.<br>Neurodevelopmental assessment metrics were corrected for age at assessment and sex. As stated for specific analysis, data were corrected for maternal education and household income.
